# Does caring for patients with advanced non-small cell lung cancer affect health-related quality of life of caregivers? A multicenter, cross-sectional study

**DOI:** 10.1186/s12889-024-17669-w

**Published:** 2024-01-18

**Authors:** Yi Yang, Liu Liu, Jia Chen, Yuying Gan, Chunxia Su, Haibo Zhang, Enwu Long, Fei Yan, Yingyao Chen

**Affiliations:** 1 School of Public Health, Fudan University, Shanghai, China; 2 National Health Commission Key Laboratory of Health Technology Assessment, Fudan University, Shanghai, China; 3 Department of Medical Oncology, The Affiliated Tumor Hospital of Nantong University &Nantong Tumor Hospital, Nantong, China; 4 Department of Respiratory Medicine, The Affiliated Hospital of Xuzhou Medical University, Xuzhou, China; 5 Department of Medical Oncology, Shanghai Pulmonary Hospital & Thoracic Cancer Institute, Tongji University School of Medicine, Shanghai, China; 6 Department of Organization and Personnel, the First Affiliated Hospital of Nanjing Medical University, Nanjing, China; 7 Department of Pharmacy, Sichuan Academy of Medical Sciences/Sichuan Provincial People’s Hospital, Sichuan, China; 8 Department of Oncology, Jiangsu Cancer Hospital & Jiangsu Institute of Cancer Research & The Affiliated Cancer Hospital of Nanjing Medical University, Nanjing, China

**Keywords:** Non-small cell lung cancer, Caregiver, Health-related quality of life, EQ-5D-5L, Supportive care

## Abstract

**Purpose:**

Patients with advanced non-small cell lung cancer (NSCLC) mostly receive essential routine care and support from informal caregivers, who usually experience poorer health-related quality of life (HRQoL). The study aimed to evaluate the HRQoL and its predictors among informal caregivers of patients with advanced NSCLC in China.

**Methods:**

We interviewed the adult caregiver population of patients with advanced NSCLC (stage IIIB~IV) in nine tertiary hospitals from multiple provinces in China between November 2020 and June 2021. The EQ-5D-5L instrument measured the HRQoL of caregivers, as analyzed by employing descriptive analysis, univariate analysis, Tobit regression, and multivariate logistic regression, and investigated the important influencing factors further.

**Results:**

A valid sample of 553 caregivers was analyzed. The mean EQ-5D-5L utility score of caregivers was 0.92 (SD = 0.14). Caregivers reported the greatest problems in mental health, with 45.39% reporting slight, moderate, severe, or extreme anxiety/depression. The potential influencing factors of HRQoL in caregivers included patients' age and cancer histology, relationship with the patients, and daily caregiving hours. Compared to other caregivers, patients' spouses had the lowest HRQoL. In addition, over six hours of caregiving per day was associated with lower HRQoL in caregivers of patients with advanced NSCLC.

**Conclusions:**

The HRQoL of caregivers for patients with advanced NSCLC was investigated for the first time in China. The informal caregivers experience decreased HRQoL, with anxiety /depression problems being reported the most. The findings of this study would provide extensive information on the HRQoL of advanced NSCLC patients' caregivers for future health-promoting self-care.

## Background

In 2020, approximately 1.8 million people died from lung cancer, the most common cancer threatening human health and lives globally [1]. In China, lung cancer remains the most incidence and the leading cause of cancer-related death for both sexes, accounting for 40% of lung cancer deaths internationally [2]. Non-small cell lung cancer (NSCLC) is the most prevalent subtype, representing roughly 85% of lung cancer cases [3, 4]. Most cases with NSCLC are diagnosed in advanced stages, accompanied by a wide range of physical, psychosocial, and practical problems owing to the poor prognosis [5]. Individuals with tumor driver genes have been gradually revealed in NSCLC patients, with notable examples such as the epidermal growth factor receptor (EGFR) and the anaplastic lymphoma kinase (ALK) [6]. Patients without EGFR or ALK driver genes may have worse prognosis than those with positive drivers, who could derive significant benefits from molecular targeted therapies, such as prolonged survival [7, 8].

During the treatment process of cancer patients, informal care provided by caregivers plays an essential part [9], which the informal caregivers (e.g., adult children, spouses, parents, or friends) usually provide cancer patients with diet preparation, emotional and psychological support, communication with healthcare providers, and other activities of daily living [10, 11]. Nevertheless, caring for cancer patients is burdensome for caregivers [12]. The experience of having cancer brings physical burden and psychological distress not only to the patient but also to the caregiver, who can sometimes be much more severely affected than the patient [5]. For instance, patients with lung cancer experience are apt to have symptoms like dyspnea, pain, persistent cough, and loss of appetite [13], which are not only typically perceived to be associated with increased anxiety, loss of function, and a decline in health-related quality of life (HRQoL) for patients [14], but also have a substantial impact on caregivers' well-being and functional capacity [13, 15]. Furthermore, compared to informal caregivers of patients with non-impaired HRQoL, individuals caring for advanced-stage cancer patients with impaired HRQoL are more likely to have significantly higher caregiving burden, poor anxiety/depression symptoms, and worse HRQoL [16].

Additionally, integrating the health-related utility data for caregivers into health economic evaluation (HEE) is increasingly critical for a comprehensive assessment of the value in health technologies to inform clinical access and healthcare insurance coverage decision-making. The healthcare interventions could potentially improve the HRQoL of both patients and their caregivers [17]. Incorporating the changes in caregivers' HRQoL (sometimes referred to as "spillover" effects) into HEE might alter the incremental quality-adjusted life years (QALYs), and thus potentially reshape the final evaluation results and relevant decision-making [18]. Consequently, a number of international HTA agencies, like the National Institute for Health and Care Excellence (NICE) in the United Kingdom, the Haute Autorité de santé (HAS) in France, the Health Information and Quality Authority in Ireland, etc., all recommend that HEE should include the direct health effects both for patients and their caregivers contributed by target healthcare interventions [18–20].

To our knowledge, several studies have focused on the health-related quality of life for patients with NSCLC [21–24], yet few researchers are concentrating on the quality of life (QoL) for their caregivers in China, especially in NSCLC. To fill this gap, we conducted this investigation to evaluate the HRQoL in caregivers of advanced NSCLC patients via the EQ-5D-5L instrument, and to further explore the influencing factors associated with caregivers' HRQoL in China.

## Methods

### Study design and population

The cross-sectional study of the caregivers for advanced NSCLC patients was conducted as a part of the Demonstration Program on Health Technology Assessment, a nationwide investigation on the patients with locally advanced or metastatic NSCLC without sensitizing EGFR and ALK alterations in China.

Caregivers refer to family members (spouse, child, cousin, etc.) who provide informal care for patients, do not possess any training in caregiving, and do not receive any economic compensation for this care task in this study. Caregivers were recruited in two steps: (i) Enrolling target patients, with the inclusion criteria embracing histologically or cytologically confirmed stage IIIB or IV NSCLC without EGFR/ALK mutation, age 18 years, and a sufficient level of physical and mental health to complete the research questionnaire independently or with the assistance of their families, and those engaging in clinical trials, illiterate or unable to read or write, and afflicted with several severe systemic illnesses were disqualified; (ii) Caregivers were enlisted concurrently with patients and were expected to be patient’s family member, comprehend the patients’ disease conditions and medical expenses, and be over 18 years of age.

### Data collection

From November 2020 to June 2021, data were collected in six general tertiary hospitals, two regional cancer centers, and one pulmonary hospital across Jiangsu, Shanghai, Fujian and Sichuan provinces in China using a mix of convenience sampling and cluster sampling. The face-to-face interviews were held using the structured questionnaire developed based on literature review and scales validated for use in our setting. Prior to the formal survey, the relevant adjustments and validations of questionnaire were made based on the pre-survey results. Before the interview, all participants were required to sign a written informed consent form to indicating their agreement to participate. After completing of each questionnaire, the interviewer undertook data supervision to ensure data completeness and consistency.

### Outcome measurements

Information collected in the structured questionnaire included three components.

1. Sociodemographic characteristics of caregivers, including gender, age, residence, material status, education, employment status, kinship with the patients, and household income, with the 2019 Chinese urban per capita disposable income CNY45,000 ($ 6,975) as the baseline option and the numbers in subsequent options increasing in multiples (CNY45,000, CNY45,000-CNY90,000, CNY90,000-CNY180,000, CNY180,000). Regarding the caregiving circumstances, the intensity (daily hours), and duration of caregiving were involved.
2. Disease-related characteristics of recipients comprising pathological type, cancer clinical stage, progression of cancer, treatment regimen, gene drive for cancer, and duration of diagnosis since diagnosis.
3. HRQoL (dependent variable) was self-assessed by caregivers using the EuroQoL-5 Dimensions (EQ-5D), which is a widely used generic preference-based HRQoL instruments [25]. It comprises two parts, a descriptive system and a 100-point visual analogue scale. The descriptive system reports on five health domains (mobility, self-care, usual activities, pain/discomfort, anxiety/depression), with each dimension having five levels: no problems, slight problems, moderate problems, severe problems, and unable to/extreme problems. The health utility values can be derived by the Chinese-specific scoring algorithm of the EQ-5D-5L developed by Luo et al, which yielded scores ranging from −0.391 to 1.000, with zero representing being dead, 1.000 indicating a state of full health, and negative scores indicating health status worse than death [26].

### Statistical analysis

For categorical variables, descriptive statistics were presented as frequency and/or percentages, and for continuous variables, as mean and standard deviation (SD). The null hypothesis of normal distribution of health state utility was rejected by the Kolmogorov–Smirnov test. To compare the differences in HRQoL among various subgroups of caregivers, the Mann–Whitney U-test or Kruskal–Wallis test was used. We performed the multivariable regression to explore the impact of sociodemographic characteristics (age, material status, education, employment status, kinship with the patients, household income, *etc*.), patients' disease-related information (pathological type, cancer clinical stage, and progression of cancer, *etc*.), and caring circumstances (daily hours, and duration of caregiving) on the EQ-5D-5L utility scores of caregivers. Tobit model was adopted since the ceiling issue (i.e., a large proportion of participants are classified as full health and with a utility score of 1.0) is evident in the utility data. In addition, this utility scores were dichotomized into good HRQoL (score > 0.85) and poor HRQoL (score ≤ 0.85), as was done in prior study [27]. Consequently, multivariate logistic regression also was employed to calculate the odds of poor HRQoL according to a variety of factors, including sociodemographic variables, disease-related features, and caring circumstances. At a p-value of 0.05, statistical significance was determined.

## Results

### Sociodemographic characteristics and caregiving circumstances of caregivers

A total of 553 principal caregivers were involved in the study. Caregivers were a mean age of 50.89 years, 58.77% were female, and 96.02% were married. The majority of them (54.43%) resided in rural areas and had not attended college (78.12%). Caregivers reported multiple occupational roles. There were private /public sector employee (15.55%), the self-employed (23.15%), farmer (14.10%) and others (15.19%). Moreover, 13.38% were jobless. The primary caregivers were the patient's spouse (49.55%) or child (40.32%). The length of time spent providing care varied within the sample, with the largest group of caregivers (46.65%) reporting more than 6 months, and 69.44% of caregivers delivering over 3 hours of care per day (Table 1).

**Table 1:** Sociodemographic characteristics and caregiving circumstances of caregivers

Characteristics	N / Mean	% / SD
Age	50.89	13.02
<45	188	34.00
45-59	213	38.52
≥60	152	27.48
Gender		
Female	325	58.77
Male	228	41.23
Residence		
Rural area	301	54.43
Urban area	252	45.57
Marital status		
Married	531	96.02
Other ^a^	22	3.98
Education		
Primary school or below	114	20.61
Junior high school	166	30.02
Senior high school	152	27.49
Undergraduate or over	121	21.88
Occupation		
private /public sector employee	86	15.55
Self-employed	128	23.15
Unemployed	74	13.38
Farmer	78	14.10
Retiree	103	18.63
Others	84	15.19
Household income ($, per year)^b^		
<6,975	248	44.85
6,975-	173	31.28
13,950-	97	17.54
≥27,900	35	6.33
Kinship with the patients		
Spouse	274	49.55
Child	223	40.32
Other	56	10.13
Intensity (daily hours)		
<3	166	30.02
3–6	129	23.33
>6	258	46.65
Duration of caregiving (months)		
<3	169	30.56
3–6	128	23.15
>6	256	46.29

*SD* Standard deviation

^a^The “Other” marital status including single, widowed, divorced, and separated

^b^ The household income was exchange from RMB to USD by the average annual exchange rate in 2021 from China Foreign Exchange Trade System (https://www.chinamoney.com.cn/chinese/bkccpr/)

### Sociodemographic and disease-related characteristics of recipients

The mean age of recipients was 63.89 years, and 77.94% were male. Among patients, 56.06% with adenocarcinoma, 36.35% with squamous cell carcinoma. Patients were on average 15.84 months after diagnosis, 65.82% of them were in stage IV. 57.32% of recipients were undergoing first-line treatment, 35.99% were receiving immunotherapy-related treatment regimens (Table 2).

**Table 2:** Sociodemographic and disease-related characteristics of recipients (patients)

Characteristics	N / Mean	% / SD
Age	63.89	9.37
<50	26	4.70
50-59	159	28.75
60-69	211	38.16
≥70	157	28.39
Gender		
Female	122	22.06
Male	431	77.94
Histology		
Squamous cell carcinoma	201	36.35
Adenocarcinoma	310	56.06
Other	42	7.59
Clinical stage		
IIIb-IIIc	189	34.18
IV	364	65.82
Duration of diagnosis since diagnosis (months)	15.84	20.98
Progression		
No	333	60.22
Yes	220	39.78
Line of treatment		
None ^a^	16	2.90
First-line	317	57.32
Second-line and above	220	39.78
Gene drive		
No	308	55.70
Yes ^b^	41	7.41
Unknown	204	36.89
Treatment regimen		
None ^c^	29	5.24
Immunotherapy-related treatments ^d^	199	35.99
Other therapies ^e^	325	58.77

*SD* Standard deviation

^a^Newly diagnosed lung cancer patients who have not yet started antitumor treatment

^b^Positive driver genes include HER2, KRAS, BRAF, BRCA1/2, ATM, TP53, RET, TET1, etc

^c^Lung cancer patients who had not started antitumor therapy at the time of the survey

^d^Immunotherapy-related treatment comprises immunotherapy monotherapy, immunotherapy plus targeted therapy, immunotherapy plus chemotherapy, and immunotherapy plus radiotherapy

^e^Other therapies group includes 11 patients receiving palliative care, 276 patients receiving chemotherapy-related treatments, and 38 patients undergoing other treatments

### Health-related quality of life (HRQoL) of Caregiver

**Fig. 1:**
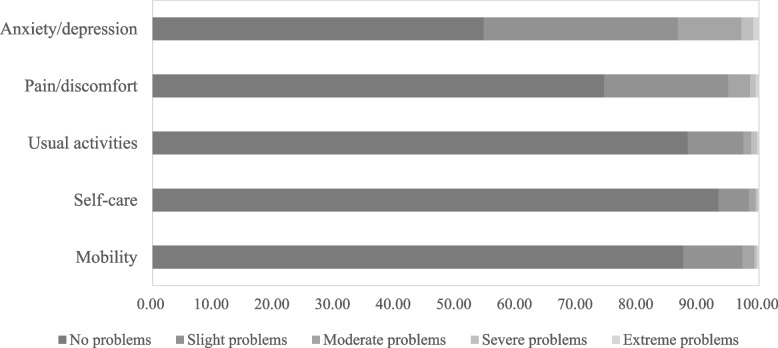
The distribution of EuroQol 5-dimensions among caregivers

The mean EQ-5D-5L utility score for caregivers was 0.92 (SD: 0.14) and the mean EQ-VAS score was 82.70 (SD: 13.83). Of the five EQ-5D-5L dimensions, 297 participants (53.70%) reported no problems on any of the five dimensions (Table 3). Figure 1 shows the proportions of the EQ-5D-5L in each dimension. Most caregivers had no problems in self-care (93.31%), usual activities (88.25%), mobility (87.52%), and pain/discomfort (74.50%). The participants reported the greatest problems in mental health, with 45.39% reporting slight, moderate, severe or extreme anxiety/depression. In addition, 25.50% of caregivers experienced problems in pain/discomfort dimension.

**Table 3:** Caregiver self-reported health captured using EQ-5D-5L

Dimensions of EQ-5D-5L	N / Mean	% / SD
Mobility problem		
No	484	87.52
Yes	69	12.48
Self-care problem		
No	516	93.31
Yes	37	6.69
Problem with Usual activities		
No	488	88.25
Yes	65	11.75
Pain/discomfort		
No	412	74.50
Yes	141	25.50
Anxiety/depression		
No	302	54.61
Yes	251	45.39
Overall/any dimension problems		
No	297	53.70
Yes	256	46.30
EQ-5D-5L health utility		
Good HRQoL (>0.85)	462	83.54
Poor HRQoL (≤0.85)	91	16.46
EQ-5D-5L health utility		
Mean (SD)	0.92	0.14
EQ-5D-5L VAS		
Mean (SD)	82.70	13.83

*SD* Standard deviation, *HRQoL* Health-related quality of life

Table 4 shows the EQ-5D-5L utility scores of each categorical variable. The EQ-5D utility scores of individuals caring the squamous cell carcinoma patients were significantly higher than those caring other subtypes of advanced NSCLC patients. Younger caregiver obtained significantly higher utility scores than older groups. Caregivers with lower education levels than junior high school and those living in the rural regions had the lowest utility scores under their respective categories. The utility scores of caregivers with an annual household income greater than $6,975 were higher than those with a yearly household income of less than $6,975. As a caregiver, Patients' children had better utility values than spouse. The difference in utility scores was statistically significant among individuals with various caregiving intensity per day.

**Table 4:** Univariate analysis of utility scores for caregivers

Characteristics	Mean	SD	*P* value
***Recipient characteristics***			
Age			0.714
<50	0.931	0.090	
50-59	0.934	0.124	
60-69	0.909	0.162	
≥70	0.918	0.143	
Gender			0.011
Female	0.908	0.122	
Male	0.923	0.149	
Histology			0.023
Squamous cell carcinoma	0.940	0.116	
Adenocarcinoma	0.913	0.149	
Other	0.869	0.202	
Clinical stage			0.099
IIIb-IIIc	0.932	0.138	
IV	0.913	0.146	
Duration of diagnosis since diagnosis (months)			0.968
<6	0.924	0.125	
6-12	0.910	0.177	
>12	0.923	0.133	
Progression			0.280
No	0.926	0.132	
Yes	0.910	0.160	
Line of treatment			0.457
None	0.936	0.066	
First-line	0.925	0.134	
Second-line and above	0.910	0.160	
Gene drive			0.985
No	0.918	0.156	
Yes	0.902	0.149	
Unknown	0.926	0.121	
Treatment regimen			0.717
None	0.944	0.056	
Immunotherapy	0.922	0.160	
Other therapies	0.916	0.139	
***Caregiver characteristics***			
Age			0.002
<45	0.933	0.157	
45-59	0.928	0.102	
≥60	0.892	0.171	
Gender			0.130
Female	0.920	0.121	
Male	0.919	0.171	
Residence			0.016
Rural area	0.912	0.146	
Urban area	0.929	0.140	
Marital status			0.470
Married	0.922	0.137	
Other	0.867	0.253	
Education			<0.001
Primary school or below	0.895	0.124	
Junior high school	0.922	0.156	
Senior high school	0.940	0.134	
Undergraduate or over	0.914	0.152	
Occupation			0.143
private /public sector employee	0.936	0.129	
Self-employed	0.930	0.129	
Unemployed	0.937	0.083	
Farmer	0.913	0.111	
Retiree	0.898	0.183	
Others	0.905	0.185	
Household income ($, per year)			0.018
<6,975	0.907	0.144	
6,975-	0.922	0.158	
13,950-	0.940	0.129	
≥27,900	0.945	0.091	
Kinship with the patients			<0.001
Spouse	0.902	0.147	
Child	0.949	0.087	
Other	0.890	0.251	
Intensity (daily hours)			0.029
<3	0.944	0.087	
3–6	0.937	0.102	
>6	0.895	0.182	
Duration of caregiving (months)			0.372
<3	0.935	0.098	
3–6	0.919	0.174	
>6	0.910	0.152	

*SD* Standard deviation

### Factors influencing caregiver of patients with advanced NSCLC health utility

The multivariate analysis (Table 5) revealed patients' histology, kinship with the patients to be significantly correlated with the health utility of caregiver. Furthermore, caregivers who care for older patients with advanced NSCLC were less likely to report good HRQoL, as were those providing care for more than six hours per day.

**Table 5:** Tobit regression and multivariate logistic analysis for EQ-5D-5L index score

Characteristics	EQ-5D-5L index score				HRQoL ^a^			
	Coefficient	(95% CI)		*P* value	OR	(95% CI)		*P* value
***Recipient characteristics***								
Age	-0.002	-0.005	0.001	0.115	0.962	0.929	0.996	0.029
Gender (Ref: Female)								
Male	0.025	-0.029	0.079	0.363	1.432	0.736	2.788	0.291
Histology (Ref: Squamous cell carcinoma)								
Adenocarcinoma	-0.023	-0.068	0.023	0.328	0.491	0.266	0.904	0.022
Other	-0.094	-0.174	-0.015	0.020	0.302	0.122	0.753	0.010
Clinical stage (Ref: IIIb-IIIc)								
IV	-0.010	-0.055	0.035	0.653	0.977	0.545	1.751	0.937
Duration of diagnosis since diagnosis (months)	0.000	-0.001	0.002	0.521	1.004	0.989	1.020	0.572
Progression (Ref: No)								
Yes	-0.010	-0.062	0.042	0.711	0.655	0.347	1.238	0.193
Treatment regimen (Ref: None)								
Immunotherapy	0.026	-0.066	0.119	0.579	0.387	0.076	1.964	0.252
Other therapies	0.021	-0.068	0.110	0.650	0.383	0.079	1.853	0.233
***Caregiver characteristics***								
Age	0.000	-0.003	0.003	0.915	0.990	0.958	1.023	0.546
Gender (Ref: Female)								
Male	0.001	-0.046	0.048	0.960	1.505	0.808	2.805	0.198
Residence (Ref: Rural area)								
Urban area	0.021	-0.029	0.071	0.399	1.612	0.824	3.153	0.163
Marital status (Ref: Married)								
Other	-0.091	-0.198	0.017	0.098	0.293	0.086	1.000	0.050
Education (Ref: Primary school or below)								
Junior high school	0.005	-0.055	0.065	0.870	1.440	0.706	2.935	0.316
Senior high school	0.032	-0.038	0.102	0.369	1.425	0.612	3.320	0.411
Undergraduate or over	-0.071	-0.155	0.013	0.099	0.453	0.162	1.268	0.132
Occupation (Ref: private / public sector employee)								
Self-employed	-0.029	-0.102	0.043	0.423	0.391	0.137	1.114	0.079
Unemployed	0.012	-0.069	0.094	0.771	1.054	0.326	3.415	0.930
Farmer	-0.048	-0.128	0.031	0.231	0.993	0.306	3.216	0.990
Retiree	-0.018	-0.094	0.058	0.638	0.683	0.244	1.909	0.467
Others	-0.037	-0.114	0.041	0.353	0.334	0.116	0.962	0.042
Household income ($, per year) (Ref: <6,975)								
6,975-	-0.004	-0.056	0.047	0.872	1.240	0.655	2.346	0.509
13,950-	0.034	-0.031	0.100	0.305	2.034	0.822	5.032	0.124
≥27,900	0.052	-0.045	0.148	0.293	0.851	0.257	2.813	0.791
Kinship with the patients (Ref: Spouse)								
Child	0.107	0.035	0.178	0.003	3.763	1.466	9.663	0.006
Other	0.021	-0.063	0.106	0.624	1.112	0.412	2.999	0.834
Intensity (daily hours) (Ref: <3)								
3–6	0.004	-0.053	0.061	0.894	0.608	0.267	1.385	0.236
>6	-0.038	-0.091	0.015	0.156	0.400	0.193	0.829	0.014
Duration of caregiving (months) (Ref: <3)								
3–6	-0.015	-0.072	0.042	0.604	1.201	0.569	2.537	0.631
>6	-0.036	-0.094	0.021	0.212	0.990	0.478	2.049	0.978
constant	1.137	0.926	1.348	0.000	465.620	23.054	9403.977	0.000

*OR* Odd ratio, *CI* Confidence interval

^a^The multivariate logistic regression was employed in which the utility score was the dependent variable dichotomized into good HRQoL (score > 0.85) and poor HRQoL (score ≤ 0.85)

## Discussion

Much work has been devoted to the prognosis and quality of life among patients with oncology, while little attention has been paid to the HRQoL for the caregiver of cancer recipients, including NSCLC. To the best of our knowledge, this is the first study to evaluate the HRQoL and factors that influence the HRQoL as well as utility among caregivers for patients with advanced NSCLC in China. Specifically, the EQ-5D-5L, the most popular preference-based instrument, was utilized. In addition, the investigation contributes to the incorporation of evidence regarding caregiver HRQoL into health economic evaluation.

Caregivers of advanced NSCLC patients has a poorer mean EQ-5D-5L utility score (0.92) than the general Chinese population (0.946) [28] and urban general Chinese population (0.957) [29]. Moreover, the mean EQ-5D-5L score of caregivers in this study is higher than what has previously been reported regarding caregivers caring for other diseases. For example, EQ-5D-5L utility ratings for primary caregivers of children with intellectual disability (ID) were estimated to be 0.8 in Australia [30]. In a separate study, informal caregivers for individuals with mild, moderate, and moderately severe Alzheimer's disease (AD) dementia had mean EQ-5D-3L index scores of 0.86, 0.85, and 0.82 respectively. With the different EQ-5D instruments (EQ-5D-3L or EQ-5D-5L) being applied, the disparities in health perception across the sample group and the difference in the caring burden of caregivers for patients with various diseases may account for the variances above. Caring for a child with ID or older with AD dementia may require more time in providing assistance or supervision for self-care, mobility, or communication than caring for cancer patients because they have behavioral disturbances or cognitive impairment associated with AD or ID [30–32].

Anxiety/depression was found to be the most commonly reported problem (45.39%) faced by caregivers of advanced NSCLC patients in this investigation, whereas pain/discomfort was the most frequently reported problem among the general Chinese population [29]. However, our analysis was consistent with prior studies based on caregiver sample caring for children with ID [30], patients with uncontrolled focal-onset seizures [33], and the individuals suffered from rare diseases [34]. Recent evidence suggests that caregivers of lung cancer patients frequently experience anxiety, depression, and psychological distress [35, 36]. Therefore, it is critical to provide available psychological therapies and social support interventions for caregivers in order to alleviate their psychological condition [37–39].

The key findings revealed that the HRQoL among caregivers of advanced NSCLC patients is associated with the patient's age, lung cancer histology, the relationship with patients, and the daily caring burden. The discovery indicated caregivers caring for older patients or individuals with partial histopathologic types, such as large cell carcinoma, had impaired HRQoL. Age could have an negative impact on HRQoL of NSCLC patients, with a higher age being associated with a poorer HRQoL [40]. The histopathologic type of lung cancer correlates with patient prognosis [41], and previous studies have demonstrated that patients with large cell carcinoma have shorter average survival time and worse prognosis than those with squamous lung cancer [42]. Consequently, caregivers may need more targeted psychological assistance and supports while caring for patients with lower HRQoL and a worse prognosis, thereby impacting their own HRQoL.

Our investigation also showed that spouses who provided informal care and caregivers with longer daily caregiving hours had poorer HRQoL, which is comparable with the findings of other studies [33]. The connection between the amount of time spent providing care and the caregiver's health may develop a negative trend [43]. Moreover, previous studies based on relatives of elderly patients with senile disorders conclusively proven spouses perceive greater stress than other relatives [44, 45]. When faced with patients' deteriorating health, continued caring demands, and the possibility of losing a loved one, spouses also likely to be distressed [46, 47]. This may explain why spouses who provide care have a worse HRQoL.

This study presents an up-to-date complement to the HRQoL evidence of caregivers for patients, especially for advanced NSCLC patients in mainland China. It will be a critical resource, providing essential measurement tools and pertinent information on influencing factors for future in-depth research into caregivers' HRQoL. Furthermore, we have utilized the EQ-5D-5L instrument to assess caregivers' health utility scores, thereby offering pertinent data for comprehensive evaluations of healthcare technologies, such as lung cancer treatments, in the future.

Compared with other studies, this work has some strengths. The comparatively large sample size for caregivers of advanced NSCLC patients in multiple medical centers of mainland China and the standardized HRQoL measurement instruments made the results more authentic and convincing. In addition to the Tobit model, a logistic regression model was used to evaluate the factors influencing HRQoL among caregivers in the good HRQoL group (utility index > 0.85) and the poor HRQoL group (utility index ≤0.85) [27], thereby enhancing the validity of the findings. However, there are several limitations in this research. First, only one HRQoL instrument (EQ-5D-5L) was employed to measure caregivers' HRQoL, which may not yield complete results. Nevertheless, the EQ-5D-5L is one of the most extensively used preference-based instruments globally. Second, given that this is a cross-sectional study, it may not be possible to identify the causal relationship between the HRQoL of caregivers and the influencing factors. Moreover, we exclusively focused on the influence factors presented in this study that may impact the HRQoL of caregivers, while other potential factors, such as the number of family members and the availability of social support, etc., were not considered. Furthermore, as only the caregivers for advanced NSCLC patients without sensitizing EGFR and ALK alterations were included in this investigation, it is restricted to generalize our findings to more settings.

## Conclusion

This study fills the gap with the absence of available evidence on the caregivers’ HRQoL of patients with advanced NSCLC in mainland China. The findings of the investigation indicate that caregivers for patients with advanced NSCLC experience worse HRQoL, with anxiety /depression problems of mental health being reported the most. Recipients’ sociodemographic and disease-related characteristics, such as age and cancer histology, as well as the relationship with patients, and the daily caring burden affects caregivers’ HRQoL. This research provides credible evidence and guidance for enhancing the HRQoL of informal caregivers for advanced NSCLC patients.

## Data Availability

All data generated or analysed during this study are included in this published article.

## Abbreviations

NSCLC: Non-small cell lung cancer
HRQoL: Health-related quality of life
HEE: Health economic evaluation
EQ-5D: EuroQoL-5 Dimensions
QALYs: Quality-adjusted life years

## References

[1] Sung H, Ferlay J, Siegel RL (2021). Global Cancer Statistics 2020: GLOBOCAN Estimates of Incidence and Mortality Worldwide for 36 Cancers in 185 Countries. CA Cancer J Clin.

[2] Cao W, Chen HD, Yu YW (2021). Changing profiles of cancer burden worldwide and in China: a secondary analysis of the global cancer statistics 2020. Chin Med J (Engl).

[3] Herbst RS, Morgensztern D, Boshoff C (2018). The biology and management of non-small cell lung cancer. Nature.

[4] Jamal-Hanjani M, Wilson GA, McGranahan N (2017). Tracking the Evolution of Non-Small-Cell Lung Cancer. N Engl J Med.

[5] Sato T, Fujisawa D, Arai D (2021). Trends of concerns from diagnosis in patients with advanced lung cancer and their family caregivers: A 2-year longitudinal study. Palliat Med.

[6] WU J, LIN Z. Non-Small cell lung cancer targeted therapy: drugs and mechanisms of drug resistance. Int J Mol Sci. 2022;23(23):15056. 10.3390/ijms232315056 PMC9738331 36499382

[7] Chen P, Liu Y, Wen Y (2022). Non-small cell lung cancer in China. Cancer Commun (Lond).

[8] Lindeman NI, Cagle PT, Aisner DL (2018). Updated Molecular Testing Guideline for the Selection of Lung Cancer Patients for Treatment With Targeted Tyrosine Kinase Inhibitors: Guideline From the College of American Pathologists, the International Association for the Study of Lung Cancer, and the Association for Molecular Pathology. Arch Pathol Lab Med.

[9] Cai J, Zhang L, Guerriere D (2021). Determinants of primary and non-primary informal care-giving to home-based palliative care cancer care-recipients in Ontario, Canada. Health Soc Care Community.

[10] Sun V, Raz DJ, Kim JY (2019). Caring for the informal cancer caregiver. Curr Opin Support Palliat Care.

[11] Litzelman K (2019). Caregiver Well-being and the Quality of Cancer Care. Semin Oncol Nurs.

[12] Hu X, Peng X, Su Y (2018). Caregiver burden among Chinese family caregivers of patients with lung cancer: A cross-sectional survey. Eur J Oncol Nurs.

[13] Grant M, Sun V, Fujinami R (2013). Family caregiver burden, skills preparedness, and quality of life in non-small cell lung cancer. Oncol Nurs Forum.

[14] Nafees B, Stafford M, Gavriel S (2008). Health state utilities for non small cell lung cancer. Health Qual Life Outcomes.

[15] Fujinami R, Otis-Green S, Klein L (2012). Quality of life of family caregivers and challenges faced in caring for patients with lung cancer. Clin J Oncol Nurs.

[16] Borges EL, Franceschini J, Costa LH (2017). Family caregiver burden: the burden of caring for lung cancer patients according to the cancer stage and patient quality of life. J Bras Pneumol.

[17] Al-Janabi H, Nicholls J, Oyebode J (2016). The need to "carer proof" healthcare decisions. BMJ.

[18] Pennington B, Eaton J, Hatswell AJ (2022). Carers' Health-Related Quality of Life in Global Health Technology Assessment: Guidance, Case Studies and Recommendations. Pharmacoeconomics.

[19] Pennington BM (2020). Inclusion of Carer Health-Related Quality of Life in National Institute for Health and Care Excellence Appraisals. Value Health.

[20] Al-Janabi H, Efstathiou N, McLoughlin C (2021). The scope of carer effects and their inclusion in decision-making: a UK-based Delphi study. BMC Health Serv Res.

[21] Shen Y, Wu B, Wang X (2018). Health state utilities in patients with advanced non-small-cell lung cancer in China [J]. J Comp Eff Res.

[22] Nafees B, Lloyd AJ, Dewilde S (2017). Health state utilities in non-small cell lung cancer: an international study. Asia Pac J Clin Oncol.

[23] Witlox WJA, Ramaekers BLT, Joore MA (2020). Health-related quality of life after prophylactic cranial irradiation for stage III non-small cell lung cancer patients: Results from the NVALT-11/DLCRG-02 phase III study. Radiother Oncol.

[24] Mazieres J, Kowalski D, Luft A (2020). Health-Related Quality of Life With Carboplatin-Paclitaxel or nab-Paclitaxel With or Without Pembrolizumab in Patients With Metastatic Squamous Non-Small-Cell Lung Cancer. J Clin Oncol.

[25] Devlin NJ, Brooks R (2017). EQ-5D and the EuroQol Group: Past, Present and Future. Appl Health Econ Health Policy.

[26] Luo N, Liu G, Li M (2017). Estimating an EQ-5D-5L Value Set for China. Value Health.

[27] Del Río Lozano M, García-Calvente MDM, Calle-Romero J, et al. Health-related quality of life in Spanish informal caregivers: gender differences and support received. Qual Life Res. 2017;26(12):3227–38. 10.1007/s11136-017-1678-2 28780713

[28] Xie S, Wu J, Xie F (2022). Population Norms for SF-6Dv2 and EQ-5D-5L in China. Appl Health Econ Health Policy.

[29] Yang Z, Busschbach J, Liu G (2018). EQ-5D-5L norms for the urban Chinese population in China. Health Qual Life Outcomes.

[30] Arora S, Goodall S, Viney R (2020). Health-related quality of life amongst primary caregivers of children with intellectual disability. J Intellect Disabil Res.

[31] Majoni M, Oremus M (2017). Does being a retired or employed caregiver affect the association between behaviours in Alzheimer's disease and caregivers' health-related quality-of-life?. BMC Res Notes.

[32] Reed C, Barrett A, Lebrec J (2017). How useful is the EQ-5D in assessing the impact of caring for people with Alzheimer's disease?. Health Qual Life Outcomes.

[33] Soare IA, Flint I, Savic N (2022). Quality of life study for caregivers of people with uncontrolled focal-onset seizures. J Med Econ.

[34] Valcárcel-Nazco C, Ramallo-Fariña Y, Linertová R, et al. Health-related quality of life and perceived burden of informal caregivers of patients with rare diseases in selected European countries. Int J Environ Res Public Health. 2022;19(13):8208. 10.3390/ijerph19138208 PMC9266302 35805867

[35] Li C, Yuan J, Huang X (2022). Correlation between depression and intimacy in lung cancer patients and their family caregivers. BMC Palliat Care.

[36] Seo YJ, Park H (2019). Factors influencing caregiver burden in families of hospitalised patients with lung cancer. J Clin Nurs.

[37] Geng HM, Chuang DM, Yang F (2018). Prevalence and determinants of depression in caregivers of cancer patients: A systematic review and meta-analysis. Medicine (Baltimore).

[38] Fekete C, Tough H, Siegrist J (2017). Health impact of objective burden, subjective burden and positive aspects of caregiving: an observational study among caregivers in Switzerland. BMJ Open.

[39] Northouse L, Williams AL, Given B (2012). Psychosocial care for family caregivers of patients with cancer. J Clin Oncol.

[40] Schulte T, Schniewind B, Walter J (2010). Age-related impairment of quality of life after lung resection for non-small cell lung cancer. Lung Cancer.

[41] Collins LG, Haines C, Perkel R (2007). Lung cancer: diagnosis and management. Am Fam Phys.

[42] Yuan G, Zhan C, Huang Y (2019). Clinical characteristics and prognosis of basaloid squamous cell carcinoma of the lung: a population-based analysis. PeerJ.

[43] Hiel L, Beenackers MA, Renders CM (2015). Providing personal informal care to older European adults: should we care about the caregivers' health?. Prev Med.

[44] Belasco AG, Sesso R (2002). Burden and quality of life of caregivers for hemodialysis patients. Am J Kidney Dis.

[45] Haley WE, Levine EG, Brown SL (1987). Psychological, social, and health consequences of caring for a relative with senile dementia. J Am Geriatr Soc.

[46] Milbury K, Badr H, Fossella F (2013). Longitudinal associations between caregiver burden and patient and spouse distress in couples coping with lung cancer. Support Care Cancer.

[47] Carmack Taylor C L, Badr H, Lee J H, et al. Lung cancer patients and their spouses: psychological and relationship functioning within 1 month of treatment initiation. Ann Behav Med. 2008;36(2):129–40. 10.1007/s12160-008-9062-7 PMC4454464 18797978

